# Impact of COVID-19 on neonatal health: Are we causing more harm than good?

**DOI:** 10.18332/ejm/120245

**Published:** 2020-04-13

**Authors:** Hazel A. Smith

**Affiliations:** 1 Trinity College Dublin, Dublin, Ireland

**Keywords:** necrotising enterocolitis, neonatal intensive care, COVID-19, breastfeeding

**Dear Editor,**

Currently, the evidence shows that pregnant women who get the novel coronavirus (COVID-19) disease are not at a higher risk of serious complications compared to healthy non-pregnant adults^1^. However, we know from studies and national reports that maternal comorbidities are increasing^2,3^ and that a sub-population of pregnant women will be at increased risk of COVID-19 complications. At the moment it is believed that COVID-19 does not cause any problems with fetal development^1^ but may increase the risk of preterm delivery. A recent rapid review^4^, of 23 studies examining COVID-19 in pregnancy, reported a preterm delivery rate of 47%. Given the predicted increase in the cases of COVID-19 in the coming weeks, it is reasonable to assume that preterm deliveries will also increase.

One of the major impacts of pre-term deliveries is necrotising enterocolitis (NEC)^5^. Neonates with NEC, depending on the severity, may need to utilise both neonatal services in maternity settings and surgical services in paediatric units.

NEC has the highest rate of gastroenterology mortality for preterm infants and is the most common reason for emergency surgery in neonates^5^. Breastmilk (either the mother’s or donor’s) is one of the most effective ways to prevent or reduce the severity of NEC^5-7^. Enabling and supporting breastfeeding provides us with an opportunity to promote neonatal health and also limit the demands on neonatal and paediatric services during a pandemic.

Yet, there are more and more stories of mothers (not suspected, suspected, or confirmed, cases of COVID-19) being allowed very limited time with their baby in the neonatal intensive care units^8,9^. We also know that donor milk is becoming harder to access^10^ and not all areas have their own milk bank, e.g. the Republic of Ireland does not have its own milk bank and relies on the United Kingdom to process and manage donor milk supplies.

The reasons for the heighten anxiety with regard to cross-infection during COVID-19 are understandable and justified. However, we need to remember that the impact of COVID-19 on health and healthcare services will last longer than the virus itself. Limiting the impact of NEC is something that we have control over. In our endeavour to do what is right to minimize the spread of COVID-19, are we increasing the risks of other potentially fatal diseases? What supports and actions have we, as healthcare providers, put in place to counteract the negative impact of COVID-19 restrictions? We may reduce mortality and morbidity from COVID-19 but are we just replacing them with other causes such as NEC? We need to ensure that what we do now is the right course of action, not just now, but for the long-term health of all neonates.

## References

[1] Royal College of Obstetricians and Gynaecologists Coronavirus infection and pregnancy.

[2] Nair M, Nelson-Piercy C, Knight M (2017). Indirect maternal deaths: UK and global perspectives. Obstetric medicine.

[3] Sullivan E, Marshall D, Li Z (2017). Maternal Mortality in High Income Countries. Journal of Paediatrics and Child Health.

[4] Mullins E, Evans D, Viner RM, O'Brien P, Morris E (2020). Coronavirus in pregnancy and delivery: rapid review. Ultrasound in Obstetrics & Gynecology.

[5] Rose AT, Patel RM (2018). A critical analysis of risk factors for necrotizing enterocolitis. Seminars in fetal & neonatal medicine.

[6] Quigley M, McGuire W (2014). Formula versus donor breast milk for feeding preterm or low birth weight infants. Cochrane Database Syst Rev.

[7] Hair AB, Peluso AM, Hawthorne KM (2016). Beyond Necrotizing Enterocolitis Prevention: Improving Outcomes with an Exclusive Human Milk-Based Diet. Breastfeed Med.

[8] Health Service Exective Hospital service disruptions and visiting restrictions (COVID-19).

[9] Bliss Coronavirus (COVID-19) Information.

[10] Bryant F (2020). US NICUs and donor milk banks brace for COVID-19. Lancet Child Adolesc Health.

